# Per-Manganate of Potassa

**Published:** 1869-11

**Authors:** 


*Per-Manganate of Potassa.*—A solution of per-manganate of potassa will be found a valuable remedy in the treatment of disease of the antrum, in the proportion of 5 parts to 100 of water. We have recently applied this solution, in the form of an injection, in several cases of disease of this cavity, and in a short time after its use was commenced the disagreeable odor was diminished and the good effects of the agent apparent. It is also valuable in ozaena and other affections similar in character.

